# Sex estimation of the sternum by automatic image processing of multi-slice computed tomography images in a Croatian population sample: a retrospective study

**DOI:** 10.3325/cmj.2019.60.237

**Published:** 2019-06

**Authors:** Ana Bedalov, Željana Bašić, Ivan Marelja, Krešimir Dolić, Krešimir Bukarica, Saša Missoni, Mario Šlaus, Dragan Primorac, Šimun Andjelinović, Ivana Kružić

**Affiliations:** 1 Faculty of Science, University of Split, Split, Croatia; 2 University Department for Forensic Sciences, University of Split, Split, Croatia; 3 Clinical Department for Diagnostic and Intervention Radiology, University Hospital Center Split, Split, Croatia; 4 Institute for Anthropological Research, Zagreb, Croatia; 5 Anthropological Center, Croatian Academy of Sciences and Arts, Zagreb, Croatia; 6 St. Catherine Specialty Hospital, Zabok/Zagreb, Croatia; 7 Eberly College of Science, The Pennsylvania State University, University Park, PA, USA; 8 University of Split, School of Medicine, Split, Croatia; 9 School of Medicine, Josip Juraj Strossmayer University of Osijek, Osijek, Croatia; 10 Faculty of Medicine, University of Rijeka, Rijeka, Croatia; 11 Henry C. Lee College of Criminal Justice and Forensic Sciences, University of New Haven, West Haven, CT, USA; 12 Faculty of Dental Medicine and Health Osijek, Josip Juraj Strossmayer University of Osijek, Osijek, Croatia; 13 Children’s Hospital Srebrnjak, Zagreb, Croatia; 14 Clinical Department for Pathology, Legal Medicine and Cytology, University Hospital Center Split, Split, Croatia; * The first two authors contributed equally.

## Abstract

**Aim:**

To determine the sexual dimorphism of the sternum with standard measurements in a contemporary Croatian population sample using multi-slice computed tomography (MSCT) and to compare the data obtained by an automatic with those obtained by a manual approach.

**Methods:**

Five sternal measurements were obtained from MSCT images of 73 men and 55 women and three sternal indices were calculated. Custom image analysis software was developed for automatic segmentation and calculation of sternal measurements. Measurements of sexual dimorphism were automatically calculated and compared with manual measurements.

**Results:**

All of the sternal measurements exhibited significant differences between men and women. The discrepancies between manual and automatic measurements ranged from 2.8% to 3.6% of the mean average values obtained with the automatic approach. The most accurate single-variable discriminant function was sternal body length (82.8%), the most accurate index was sternal area (89.1%), and the discriminant function using three variables was manubrium width, sternal body length, and sternal body width (90.6%).

**Conclusion:**

Sternal measurements are a reliable sex indicator and can be used in forensic casework. Computer-aided measurement methods can accelerate sex estimation and improve its precision and accuracy.

In forensic anthropology, human bones can provide important information for estimating sex, age-at-death, and stature of deceased individuals. After ancestry estimation, sex estimation is one of the first steps leading to the identification of an individual. However, the methods used cannot be applied in every single case, especially when dealing with fragmented skeletal remains. Sex estimation can be performed by using three methods: morphologic, osteometric (1), and DNA analysis (2). Osteometric measurements use is recommended if the population affinity of a specimen is known, but this approach has the important limitation of population specificity, especially in forensic casework and mass disasters victim identification. The major drawback of morphologic methods is their subjectivity and lack of statistical approach (3), while the drawbacks of DNA analysis include inhibition, degradation, and contamination, along with the higher cost and labor intensiveness (4). Also, the analysis of amelogenin, which is included in the Combined DNA Index System, can misinterpret the individual’s sex (5). Both morphological and osteometric methods could be improved by using data from well documented physical osteological collections, and more recently from virtual osteological collections that use radiographic images from individuals of a known biological profile. Radiological techniques of bone structure allow us to detect sex-, age-, and stature-related morphological and osteometric features and calculate the functions for biological profile estimation (6). In this respect, computed tomography (CT) was successfully used for virtual bone measurement in stature and sex determination (7,8). Besides other anatomical regions, the analysis also included the thorax – only the sternum or the whole rib cage (9-11). The measurements included size, shape (12), and kinematics of the rib cage (13), as well as thoracic vertebrae (14). Although these studies represent a considerable part of the recent literature, none of them was conducted on the Croatian population. Since the population specificity of body measurements requires constructing the standards for each population, the aim of this study was to verify the applicability for sex estimation of standard osteometric sternum measurements obtained in a Croatian population sample on the basis of multi-slice computerized tomography (MSCT) two-dimensional (2D) projection images. Also, to automatize time-consuming radiologic image analyses currently being performed manually by experienced observers and to increase the precision of osteometric measures, we developed a novel machine-learning based MSCT image processing analysis (automatic approach). To validate the automatic approach and assess its precision, we compared five standard osteometric measurements obtained by the automatic approach with the values obtained manually by experienced radiologists and anthropologists.

## Materials and methods

The images were sampled from the Virtual MSCT Database Split (University Department of Forensic Sciences), which was founded in 2017. The founding of the database and our analysis were approved by the Ethics Committee of the University Department of Forensic Sciences (024-04/17-03/00026;2181-227-05-12-17-0003) and University Hospital Centre Split 500-03/17-01/56;2181-147-01/06/M.S.-17-2). According to the Declaration of Helsinki, all data were anonymized before the study (15).

The sample consisted of 55 women and 73 men, who were imaged at the Clinical Department for Diagnostic and Intervention Radiology at the University Hospital Center Split with Somatom Sensation 16 (Siemens, Erlangen, Germany). The following parameters were used for imaging: 16 lines of detectors, the voltage of 120 kV, mAs- 62, spatial resolution 30 lp/mm (line pair), and the layer thickness of 1-3 mm, according to the standard protocol for thorax imaging. MSCT images were collected only from the individuals who were imaged without contrast or sedation. The individuals' age range was 18-83 years (women 62.6 ± 10.7; men 61.3 ± 15.2). To avoid the effect of aging on sternal measurements, the images were taken only from the patients without pathological and traumatic changes that could have affected the investigated measurements.

Based on previous data indicating the potential relationship between the manubrium and sternal body size and sex (16), we identified the following five measurements for osteometric analyses: manubrium length (M), manubrium width (MW), sternal body length (B), and sternal body width at level of the incisurae costales 1 (CSW1) and 2 (CSW2). The measurements are defined as follows:

“Manubrium length (M): the longest distance from the midpoint of the manubrium (between the incisura jugularis and incisura clavicularis) and the manubriosternal junction.

Manubrium width (MW): the width at the level of the line passing from the incisura costalis 1 midpoint on the right and left.

Sternal body length (B): the longest distance between the manubriosternal junction and mesoxiphoid junction.

Corpus sterni width at level of the incisurae costales 1 (CSW1): sternal width at the level of the line passing from the incisurae costales 2 and 3 midpoint on the right and left.

Corpus sterni width at level of the incisurae costales 2 (CSW2): sternal width at the level of the line passing from the incisurae costales 4 and 5 midpoint on the right and left (16).”

Manual measurements of osteometric variables were performed by experienced radiologists and anthropologists with Osirix v.3.9.4 (Pixmeo, Geneva, Switzerland). In the first step, the manubrium and sternal body were manually arranged in a plane so that 2D images of all the standard measurements can be obtained. Experienced radiologists and anthropologists determined the position of each end-point of each standard measure. Distance calculation in pixels, and all other osteometric values, were reported in centimeters. The images were exported in TIF format and grouped by sex and measure.

**Figure 1:**
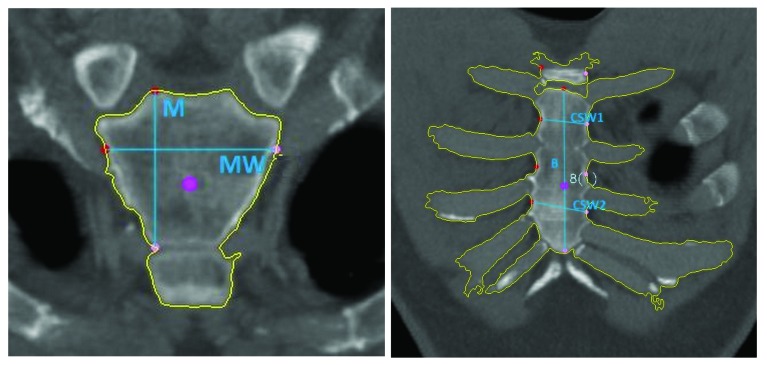
Identification of contours and determination of manubrium (left panel) and sternal body (right panel) osteometric measurements by the automatic approach. Segmentation contours (yellow lines) and contour’s body center-of-mass position (central pink dots).

In the automatic approach, morphometric MSCT images were processed with the image processing protocol BONE-SEGM, one of the modules of a larger machine learning-based image processing suite KARMEN v.1.2. – R&D studio for smart structure recognition in images (Bedalov d.o.o., Kaštel Sućurac, Croatia, *http://bedalov.org/karmen*). The protocol is carried out in two main phases: segmentation and osteometric measurements calculation. In the segmentation phase, the potential contours of the segmented manubrium and sternal body are identified by using advanced algorithms of image analysis. This phase included machine learning (iterative procedure of internal checks of variables) at three levels: 1) fitting, 2) interpolation, and 3) verification. Image processing included the following algorithms: adaptive threshold on local areas, topological potential analysis for removing poor border/segment candidates, structural connection modeling reconstructing breaks along borders, border contour reconstruction, and classification procedure to eliminate the contours with unsuitable bone characteristics. Outputs of the segmentation phase were contours for each bone. These contours were then submitted to the second phase, where five standard osteometric variables were measured by finding landmark configuration consisting of start- and end-points by using the algorithm of local extremes and in sections (Figure 1). Additionally, the stack of all standard measurements was reduced through clustering and finding the best candidate in each cluster. A training protocol was performed to optimize the parameters for each phase step to figure out the best local minimum inside the contour. For each bone, the total image processing time of each MSCT scan image using currently standard Windows-based configuration (Desktop PC with Intel Core i5 Processor [4 × 2.0 GHz] and 8 GB DDR4 RAM) was less than one second.

For the obtained measurements, three indices were calculated: sternal index (SI), sternal area (SA), and the combined length of the manubrium and body (CL). Sternal index was calculated as the division of M by B multiplied by 100; sternal area was calculated by multiplying the sum of M and B with the sum of MW, CSW1, and CSW2 divided by three, and; combined length of the manubrium and body was calculated as the sum of M and B (16-18).

### Statistical analysis

Normality of distribution was tested with Shapiro-Wilk test. Osteometric data are reported in centimeters and expressed as means and standard deviations. Manually and automatically obtained data were compared with the two-tailed pairwise *t* test, while the estimation of sex differences also included the generation of discriminant functions. The analyses were performed in SPSS, version 17 (SPSS Inc., Chicago, IL, USA). The statistical significance level was set at *P* < 0.01. The precision of the discriminant functions and sectioning points was calculated on the basis of standardized and unstandardized coefficients, as well as the structural matrix. The accuracy of the discriminant functions was evaluated in the original and cross-validated sample (only cross-validated results using leave-one-out rule are shown).

## Results

### Methods comparison

The values of five osteometric measurements were obtained from 126 CT scans. We compared the automatically obtained with the manually obtained values (Table 1). For each CT scan and each osteometric measurement, the distance was measured as the absolute length difference between two approaches. The discrepancy was measured as the percentage ratio of the mean distance and the mean value for each osteometric measure.

**Table 1:** Comparison of manual and automatic measurements (N = 126)

Osteometric measurement*	Manual (cm)		Automatic (cm)		Mean distance (cm)	Percentage of discrepancy
**mean**	**SD**	**mean**	**SD**
**CSW1**	2.65	0.36	2.66	0.37	0.07	2.8
**CSW2**	3.12	0.56	3.11	0.59	0.11	3.6
**B**	9.46	1.44	9.46	1.60	0.33	3.5
**MW**	5.63	0.53	5.63	0.53	0.16	2.8
**M**	5.23	0.53	5.25	0.57	0.17	3.1

*CSW1 – sternal body width at level of the incisurae costales 1; CSW2 – sternal body width at level of the incisurae costales 2; B – sternal body length; MW – manubrium width; M – manubrium length; SD – standard deviation.

**Figure 2:**
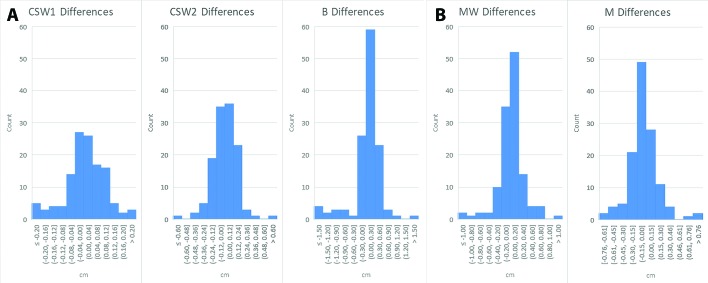
Distribution of pairwise differences between manual and automatic measurements for sternal body length (B) and sternal body width at level of the incisurae costales 1 (CSW1) and 2 (CSW2) (**A**) and manubrium length (M), manubrium width (MW) (**B**). Each panel has the same number of bins (n = 11), producing different bin widths across osteometric measurements.

There were no significant differences between the results of both approaches for any of the five osteometric measurements (pairwise *t* test, n = 126 for CSW1, CSW2, and B, and n = 126 for MW and MW1, all *P* > 0.18 or more). The percentage of discrepancies was well below 5% for all measures. CSW1 and MW were the most consistent measures, with 2.8% of discrepancy, corresponding to about 0.07 cm and 0.16 cm distances between the two approaches. CSW2 was the least consistent measure, with 3.6% discrepancy, corresponding to 0.11 cm distance difference. To assess the normality of distribution of osteometric measurements, we also calculated pairwise differences of the lengths obtained with the two approaches. The distributions of pairwise differences between the approaches for each of the five osteometric measurements was normal, with the smallest differences being the most frequent (Figure 2). Moreover, the frequency of differences rapidly decreased with the magnitude of differences, particularly for B, M, and MW. The smallest bin widths were observed for CSW1 histogram, and the largest bin widths for sternal body histogram, reflecting their variable length ranges.

**Figure 3:**
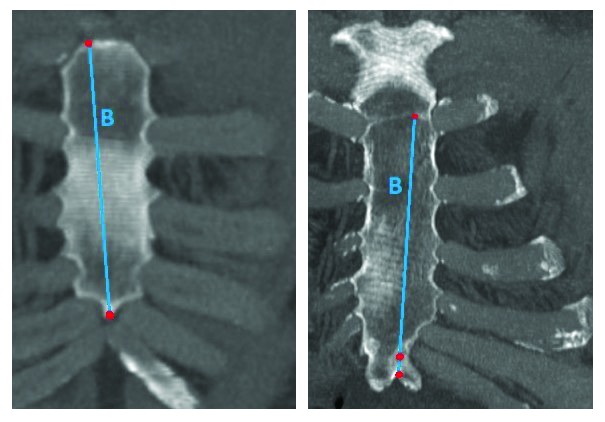
The multi-slice computed tomography (MSCT) images of two sternal bodies showing exemplary situations for landmark recognition. MSCT scan on the left allowed easy identification of landmarks for sternal body length (B), while the right one has fewer clear contours.

In absolute terms, the largest differences between manual and automatic approach were found for B. Apart from being the longest of all five measures, it was the variable for which it was most difficult to precisely locate the landmarks, both in the manual and automatic approach (Figure 3). However, the percentage of discrepancy for B (3.5%) was still comparable with the percentages of other measurements (Table 1).

### Sex estimation using sternal measurements

**Figure 4:**
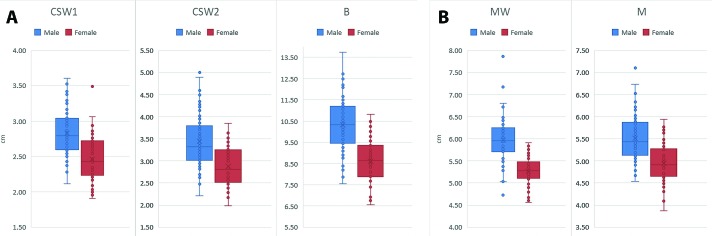
Sexual dimorphism of sternal body length (B) and sternal body width at level of the incisurae costales 1 (CSW1) and 2 (CSW2) (**A**) and manubrium length (M), manubrium width (MW) (**B**). Male – blue, female – red.

Given that the individuals’ sex was known, we determined the possibility to estimate the sex on the basis of five osteometric sternal measures obtained with the automatic approach. Men had significantly greater lengths of all five osteometric measurements (*t* test, n [male] = 72, n [female] = 54, all *P* < 0.001) (Figure 4).

The calculated discriminant functions for a combination of three measurements, single measurement, and indices yielded an overall accuracy ranging from 63.3% to 90.6% (Table 2). The most accurate single-variable discriminant function was sternal body length (82.8%), the most accurate index was sternal area (89.1%), and the discriminant function using three variables was manubrium width, sternal body length, and sternal body width (90.6%). *Post-ho*c power analysis showed that (α = 0.05) the observed power was 1 (*P* < 0.001) for all five variables.

**Table 2:** Discriminant functions for sternal measurements and indices with classification rates

Discriminant function*	Sectioning point (>males,<females)	Classification rates, %		
		men	women	overall
0.965 × MW + 0.712 × B + 1.793 × CSW1 - 17.123	-0.183	89.0	92.7	90.6
M × 2.224 - 11.653	0.07	64.4	72.7	68
MW × 2.401 - 13.642	-0.1195	78.1	80	78.9
B × 1.008 - 9.676	-0.1185	83.6	81.8	82.8
CSW1 × 3.209 - 8.590	-0.0915	72.6	74.5	73.4
CSW2 × 1.920 - 6.117	-0.072	65.8	69.1	67.2
CL × 0.934 - 13.991	-0.1405	84.9	83.6	84.4
SI × 0.125 - 6.912	0.046	69.9	54.5	63.3
SA × 0.069 - 4.019	-0.088	84.9	94.5	89.1

*CSW1 – sternal body width at level of the incisurae costales 1; CSW2 – sternal body width at level of the incisurae costales 2; B – sternal body length; MW – manubrium width; M – manubrium length; CL – combined length of the manubrium and body; SI – sternal index; SA – sternal area.

## Discussion

This study showed that sternal measurements were a reliable sex indicator in the Croatian population and that the automatic measurement method was a valuable tool for future research. To the best of our knowledge, no study so far has assessed sexual dimorphism of the sternum in either archaeological or modern Croatian populations. This is important since results from other populations cannot be applied to the Croatian population because of population specificity, ie, the difference between populations in body size due genetic, social, or environmental factors (19-21). Also, other studies focused on long bones, the skull, and the pelvis, rather than the sternum.

When applying bone measurements for sex estimation, one must consider the degree of bone preservation after the exposure to taphonomic conditions. It seems that the sternum is usually a relatively well preserved bone, making it an important candidate for identification purposes (22,23). For example, the sternum preservation in the sample of Bongiovanni and Spradley was around 60% (24). Also, it seems that single bones can be reliably used for sex estimation – the single measurement of the tibia was a more reliable sex indicator than the multivariate analysis of the cranium (25).

As male bones are usually larger and more robust than female bones (3,26,27), all of the sternal measurements were significantly larger in men than in women. The best function for discriminating between men and women was the combination of three measurements (manubrium width, sternal body length, and sternal body width), which provided 90.6% overall accuracy. The most reliable single measurement was sternal body length, with a classification rate of 82.8%, which is higher than in similar studies (16). Compared with the results of sternal measurements analysis in the modern Turkish population, this study showed higher accuracy of sex estimation (84.7% vs 90.6%) for the combination of measurements and for the sternal area (81.8% vs 89.1%). In fact, all the measurements and indices obtained in this study, that is, manubrium length (16,19), sternal body length (16,19), sternal area (16-18), and combined length, had higher accuracies than those obtained in all the previous studies (16,17,19,28), except one (29). Thus, the sternum is shown to be reliable sex estimator in the modern Croatian population, especially when the combination of three measurements: manubrium width, sternal body length, and sternal body width, is used.

Several other studies also automatically determined osteometric measurements reconstructed from either CT scans or virtual 3D scanning. For example, Inamori-Kawamoto et al (30) applied CT morphometry of the calcaneus and talus for sex estimation in the Japanese population. They obtained between 71% and 88% accuracy, depending on subjects’ age and CT measures used. Hishmat et al (31) analyzed the efficacy of automatic approach image processing of CT scans of the femur bones and found that men and women significantly differed in the femur mass volume/body height ratio. While these studies performed the virtual 3D reconstruction of human bones for volumetric analyses, our approach was based on automatized, machine learning-based image-processing algorithms for length determination of five selected osteometric measurements directly from 2D MSCT scans. While 2D data from planar MSCT scans may represent a less sensitive approach for sex estimation compared with 3D volumetric reconstruction, we believe that our processing protocol provides reliable sex estimators. The high accuracy of sex estimation using discriminant functions of sternal measurements in our sample confirms our approach as a practical option for forensic and anthropological analyses of the sternum.

Fully automated recognition of bone segments on MSCT-extracted 2D images and calculations of standardized measures could accelerate the anthropologic and forensic analysis and make it more precise by avoiding the observer errors. This study showed no significant differences between the manual and automatic methods. In addition, the automatic method significantly reduced the data acquisition and analysis time for at least two orders of magnitude. This is especially important in larger samples, where observers’ efficiency and precision decrease while software efficiency increases. Data acquisition (once the MSCT images are already obtained) and analysis time is a very important benchmark for comparing methods. Deep learning method automatically recognizes the bone segment and measures its length in a significantly shorter time than experienced observers are able to do using the manual method. The time needed to run the training for efficient segmentation parameters of bone contours depends on the number of representative images from the sample. For BONE-SEGM algorithm, it takes 20-30 minutes per bone to run the training on optimal 35 images in order to reach 98% efficiency. After training and setting the optimal parameters, it takes the algorithm 0.5 sec per image to perform segmentation, classification, and length measurements, and export the position and distances information for the rest of the images in the table form. Therefore, the algorithm can process 130 images of the same bone in maximum 30 min (training), plus 0.5 sec for automatic recognition of the rest of the 100 images.

On the other hand, for an experienced observer it takes several steps in Osirix to manually mark the distances for standard measures on the bone sample. He or she has to open the file, choose the command for measurement between two points on the image, manually place the cursor on the recognized positions for 2 or 3 distant measures, export the data in (pix/cm), and export the coordinates of the chosen positions on image, importing the data for each image/bone into the table). The time necessary for the experienced observer to manually process each image ranges from 2-4 min, with prolonged time as the observer gets tired. Also, the observer’s efficiency and precision decrease with time. Therefore, the experienced observer needs on average 6.45 h (about 23 000 sec) to process 130 images, compared with the software’s 500 sec. In addition, the discrepancy between the methods exponentially grows with a larger number of data.

As some sternal measurement have shown to be reliable for sex estimation, the further step would be to test some other sternal measurements that can be important for sex estimation. The sample should also be enlarged and complemented with subjects from other Croatian regions. The limitation of this study is the retrospective nature of the study, which can affect the sample variability. In conclusion, this study showed that some of sternal measurements are reliable sex indicators, and that forensic anthropology can benefit from automatic determination of measurements of interest. Further development of these methods, as well as enlarging the database, can help us develop other sternum-based sex estimation functions and standard measurements for other potentially useful bones.
